# Expression of secreted frizzled-related proteins in acute aortic dissection patients and the effects on prognosis

**DOI:** 10.3389/fcvm.2023.1139122

**Published:** 2023-12-22

**Authors:** Huangtai Miao, Xiaoying Li, Ying Liang, Hao Tang, Zihao Song, Shaoping Nie

**Affiliations:** ^1^ Center for Cononary Artery Disease, Beijing Anzhen Hospital, Capital Medical University, Beijing, China; ^2^ Department of Health Care for Cadres, Beijing Jishuitan Hospital, Beijing, China; ^3^ Emergency & Critical Care Center, Beijing Anzhen Hospital, Capital Medical University, Beijing, China; ^4^ School of Basic Medicine Sciences, Capital Medical University, Beijing, China

**Keywords:** acute aortic dissection, secreted frizzled-related proteins, follow-up, endpoint events, prognosis

## Abstract

**Background:**

Secreted frizzled-related proteins (SFRPs) were reported to be involved in cardiovascular diseases. This study aimed to observe plasma SFRP levels in acute aortic dissection (AD) patients and the effects of SFRP expression on AD prognosis.

**Methods:**

Plasma levels of SFRP1, SFRP2, SFRP3, SFRP4, and SFRP5 were measured in AD patients and non-AD (NAD) patients. The end-point events information of AD patients, including all-cause death and various clinical complications due to aortic dissection, was collected during a 36-month follow-up.

**Results:**

The SFRP1, SFRP2, SFRP3, and SFRP4 levels were increased in AD patients compared with those in NAD patients, while the SFRP5 concentrations were decreased. No differences in any of the SFRP levels were observed between the type A group and the type B group. The AD patients with end-point events exhibited higher SFRP1, SFRP2, SFRP3, and SFRP4 concentrations but lower SFRP5 levels than the patients without end-point events. In addition, the AD patients were divided into a high group and a low group based on the median SFRP levels, and Kaplan-Meier analysis revealed that the AD patients with high SFRP1, SFRP2, SFRP4, or SFRP5 levels had a better prognosis than those with low levels. However, the AD patients with high SFRP3 levels exhibited the opposite trends. The binary logistic regression analysis found that SFRP1, SFRP2, SFRP4, and SFRP5 were all negatively correlated with the occurrence of end-point events, while SFRP3 was positively correlated with its occurrence.

**Conclusions:**

SFRP levels are all changed in acute AD, which may affect the prognosis of AD patients. SFRPs may be a target to improve the prognosis of AD.

## Introduction

Aortic dissection (AD) is a complex clinical disease characterized by different degrees of aortic intimal tears, accompanied by the formation of a true lumen, a false lumen, and intermural hematoma. At present, aortic replacement and thoracic endovascular aortic repair (TEVAR) are the main treatments for AD (1, 2). However, both surgical methods have certain limitations, postoperative complications such as acute renal failure, cardiac tamponade, recurrent AD and so on, which seriously affects the patient's prognosis and the overall prognosis is still poor (2, 3). Therefore, finding suitable methods to reduce the occurrence of complications is crucial for improving the prognosis of AD.

Secreted frizzled-related proteins (SFRPs) are an important class of adipokines. At present, a total of 5 members have been identified, and they are named SFRP1 to SFRP5. Both can competitively bind to SFRP receptors in the Wnt pathway and inhibit the function and activity of the Wnt pathway (4). Therefore, SFRPs are considered important residual inhibitors of the Wnt pathway. SFRPS can participate in a variety of biological activities by inhibiting the Wnt pathway, and these pathways include tissue and organ development, adipocyte differentiation, lipid metabolism, inflammatory response and oxidative stress (4–7).

Numerous studies have confirmed that all SFRP members are involved in the cardiovascular disease process (7). SFRP1 has been reported to promote angiogenesis and reduce myocardial infarction size and cardiac rupture in animal studies (8, 9). SFRP2 was found to induce endothelial tube formation, reduce myocardial infarction area and improve cardiac dysfunction (10, 11). Clinical studies have found that although SFRP3 can increase the survival rate of patients with chronic heart failure caused by ischemic cardiomyopathy; an elevated SFRP3 level means that the prognosis of patients with acute coronary syndrome is poor (12, 13). Animal studies suggest that SFRP4 inhibits the progression of atherosclerosis and alleviates ischemia-induced cardiomyocyte apoptosis and heart failure in ischemic cardiomyopathy (14, 15). SFRP5 expression is decreased in coronary heart disease patients, and a high SFRP5 level indicates a good prognosis (16). Nevertheless, the role of SFRPs in AD is unknown. The aims of this study were to examine SFRP expression in acute AD and to observe the effect of SFRP expression on prognosis.

## Materials and methods

### Study population, inclusion criteria and exclusion criteria

Consecutive patients with chest pain (*n* = 352) who were hospitalized in Beijing Anzhen Hospital from December 2019 to January 2020 were included in this study. Among them, patients (*n* = 135) who had a history of diseases that may affect the follow-up results were excluded from this study, similar to in our previous article (17), and these disease included coronary artery disease (CAD, *n* = 35), dilated cardiomyopathy (DCM, *n* = 19), valvular heart disease (VHD, *n* = 9), chronic heart failure (CHF, *n* = 21), cancers (*n* = 11), peripheral arterial disease (PAD, *n* = 17), chronic obstructive pulmonary disease (COPD, *n* = 15), and Marfan syndrome (*n* = 8). The remaining patients (*n* = 217) received aortic computed tomography angiography (CTA) and were divided into an AD group (*n* = 157) and a non-AD (NAD, *n* = 60) group according to their results. The AD group was further divided into a type A group and a type B group based on whether the tear site accumulated in the aortic arch. Most AD patients underwent aortic replacement (*n* = 75) or thoracic endovascular aortic repair (TEVAR, *n* = 68), and a small number of patients were not able to tolerate surgical treatment. A few patients (14) were not available or could not tolerate surgery. This study was approved by the Ethics Committee of Beijing Anzhen Hospital, Capital Medical University (approve No. 201912104C). All participants were informed of the basic procedure of the study and signed informed consent forms.

### Information collection

Venous blood from each subject was collected in vacutainer tubes after the subjects were admitted, the samples were sent to the relevant testing center for further testing, and information on random glucose (Glu), total cholesterol (TC), high-density lipoprotein cholesterol (LDL-C), white blood cells (WBCs), creatinine (TC), D-dimer, cTnI, NT-proBNP, and C-reactive protein (CRP) was recorded. The admission records of the subjects were also required to be completed after admission, and information on sex, age, body mass index (BMI), hypertension, type 2 diabetes mellitus (T2DM), systolic blood pressure (SBP), and diastolic blood pressure (DBP) were obtained. The aortic lesions and surgical information were obtained from the aortic CTA results and surgical records and are listed in Table 1.

**Table 1:** Perioperative characteristics in aortic dissection patients.

Characteristic	Stanford A	Stanford B
Surgery		
Replacement of aorta (*n*, %)	71 (83.5)	4 (5.6)
TEVAR (*n*, %)	6 (7.1)	62 (86.1)
Do not underwent surgery (*n*, %)	8 (9.4)	6 (8.3)
Operation time (hours)		
Replacement of aorta	4.2 (2.9, 5.3)	4.1 (2.7, 5.2)
TEVAR	2.6 ± 1.4	2.3 ± 1.1
Time from attack to operation	7.2 (5.9, 11.3)	6.9 (5.5, 10.7)
Aortic tear information		
Tear length (mm)	139 ± 64	155 ± 77
Aorta coverage distance (mm)	150 ± 70	171 ± 89
Hospitalization time (days)	13.8 ± 4.7	7.9 ± 2.1

### Blood sample collection and SFRP detection

Blood samples from each subject were collected in vacutainer tubes containing sodium heparin. The plasma samples obtained were centrifuged at 4,000 × g for 20 min and then stored at −80°C until the beginning of the experiments. The plasma levels of SFRP1 (Yuanmu Biological Technology, China), SFRP2 (USCN Life Science Inc., USA), SFRP3 (Aviscera Bioscience, USA), SFRP4 (MyBioSource, San Diego, USA), and SFRP5 (USCN Life Science Inc., USA) were detected using enzyme-linked immunosorbent assay (ELISA) according to the manufacturer's instructions.

### Follow-up and end-point events

All patients were asked to provide at least 2 commonly used telephone numbers at the time of admission to complete the admission chart and to ensure the smooth progress of follow-up. At the time of discharge, the local patients were required to be followed up monthly in the outpatient clinic, and the nonlocal patients were required to be followed up by telephone once a month. For patients who were not reviewed in time, we made inquiries and reminders by pre-leaving telephone numbers. The follow-up began after the patient was admitted, and the maximum duration did not exceed 36 months. The follow-up period for these patients was 30 ± 7.2 months. Information on end-point events, including all-cause death and various clinical complications due to aortic dissection, was collected during the follow-up. The occurrence and time of end-point events are shown in Table 2.

**Table 2:** Summary of endpoint events in aortic dissection patients.

Months	Numbers	Endpoint events
1	9	Death
	2	Acute left heart failure
	1	Acute renal failure
	1	Cardiac tamponade
	1	Acute abdominal pain
	1	Paraplegia
2	3	Death
	1	Acute left heart failure
	1	Acute renal failure
3	1	Death
4	1	Death
7	1	Ischemia and dysfunction of lower limbs
11	1	Recurrent aortic dissection
12	1	Death
15	1	Acute ischemic stroke
17	1	Recurrent aortic dissection
18	1	Ischemia and dysfunction of lower limbs
20	1	Death
22	1	Recurrent aortic dissection
26	1	Acute renal failure

### Statistical analyses

Data in this study were analyzed using GraphPad Prism 7 software. Continuous variables with normal distribution are expressed as the mean ± SD and were compared by Student's *t*-test, Continuous variables with abnormal distributions were expressed as the median (lower quartile to upper quartile) and compared by the nonparametric test. Categorical variables are presented as percentages and were analyzed by a chi-squared test. The Kaplan–Meier method and log-rank test were used to compute the outcome of AD patients. Binary logistic regression analysis was performed after the factors were adjusted to determine whether SFRPs were associated with the occurrence of end-point events. *p* < 0.05 was considered significant.

## Results

### Comparison of clinical characteristics between NAD patients and AD patients

Compared with the NAD group, a higher incidence rate of smoking and hypertension and elevated values of SBP, WBC, D-dimer, NT-proBNP, and CRP were observed in the AD group. No differences in other characteristics, including sex, BMI, T2DM, DBP, Glu, TC, and LDL-C, were observed between these 2 groups. In addition, higher age and CREA, as well as lower hypertension incidence rate, WBC, D-dimer, cTnI, NT-proBNP and CRP were found in the type B group compared with the type A group. The characteristics for each group are listed in Table 3.

**Table 3:** Clinical characteristics of NAD patients and AD patients.

Characteristics	NAD	AD		
		Total	Type A	Type B
Male (*n*, %)	46 (76.7)	128 (81.5)	71 (83.5)	57 (79.2)
Age (years)	48.3 ± 8.0	52.5 ± 11.7*	46.8 ± 9.2	59.2 ± 10.4^†^
Smoking (*n*, %)	18 (30.0)	52 (61.2)*	29 (34.1)	23 (31.9)
BMI (kg/m^2^)	24.4 ± 2.4	24.4 ± 2.5	24.2 ± 2.6	24.7 ± 2.2
Hypertension (*n*, %)	10 (17.0)	77 (77.3)*	77 (90.6)	46 (63.9)^†^
T2DM (*n*, %)	4 (6.7)	11 (7.0)	6 (7.1)	5 (6.9)
SBP (mmHg)	127 ± 15	141 ± 19*	141 ± 16	140 ± 22
DBP (mmHg)	82 ± 10	85 ± 11	84 ± 11	87 ± 11
Glu (mmol/L)	5.6 (5.0, 6.0)	6.0 (4.9, 6.4)	6.1 (5.0, 6.6)	5.9 (4.9, 6.3)
TC (mmol/L)	4.5 (4.0, 4.8)	4.5 (4.0, 4.9)	4.5 (4.2, 4.8)	4.5 (4.0, 5.0)
LDL-C (mmol/L)	2.4 (2.0, 2.7)	2.3 (1.9, 3.0)	2.1 (1.6, 2.8)	2.5 (2.2, 3.0)
WBC (×10^9^/L)	5.6 (4.06 6.3)	7.2 (5.6, 9.4)*	7.5 (5.8, 11.9)	6.7 (5.4, 8.1)^†^
CREA (μmol/L)	57 (49, 63)	68 (57, 87)*	70 (59, 87)	67 (57, 88)^†^
D-dimer (ng/ml)	265 (217, 446)	1,652 (696, 3,127)*	1,972 (772, 4,892)	1,390 (624, 2,349)^†^
cTnI (ng/ml)	–	0.12 (0, 2.58)	2.35 (0.62, 6.73)	0.01 (0, 0.04)^†^
NT-pro BNP (pg/ml)	56 ± 25	244 ± 229*	316 ± 251	159 ± 164
CRP (mg/L)	2.0 (1.2, 2.7)	6.3 (3.6, 13.5)*	7.3 (3.7, 18.7)	5.2 (3.6, 9.0)^†^

* *p* < 0.05 vs. the NAD group.

^†^ *p* < 0.05 vs. the type A AD group.

### Expression levels of SFRPs in acute AD patients

**Figure 1:**
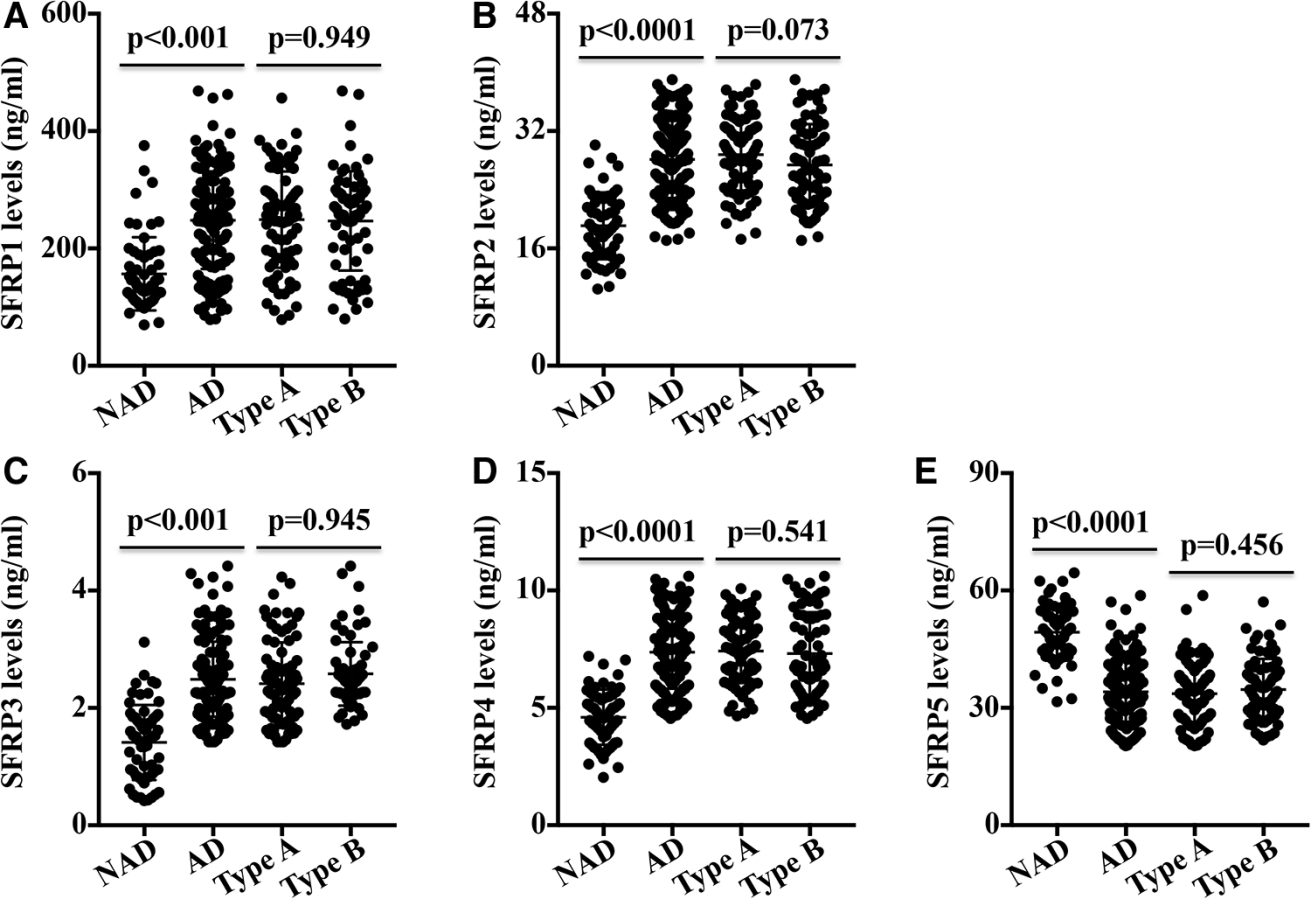
Circulating SFRP levels in NAD and AD patients were measured. (**A**) SFRP1, (**B**) SFRP2, (**C**) SFRP3, (**D**) SFRP4, and (**E**). The levels of SFRP5 in the NAD group, AD group, type A group, and type B group was measured.

The ELISA results showed that the acute AD patients exhibited higher plasma SFRP1, SFRP2, SFRP3, and SFRP4 levels and lower SFRP5 levels than the levels in the NAD group (Figures 1A–E). No differences in any SFRP were observed between the type A and type B groups (Figures 1A–E).

### Plasma SFRP concentrations in AD patients with or without endpoint events

**Figure 2:**
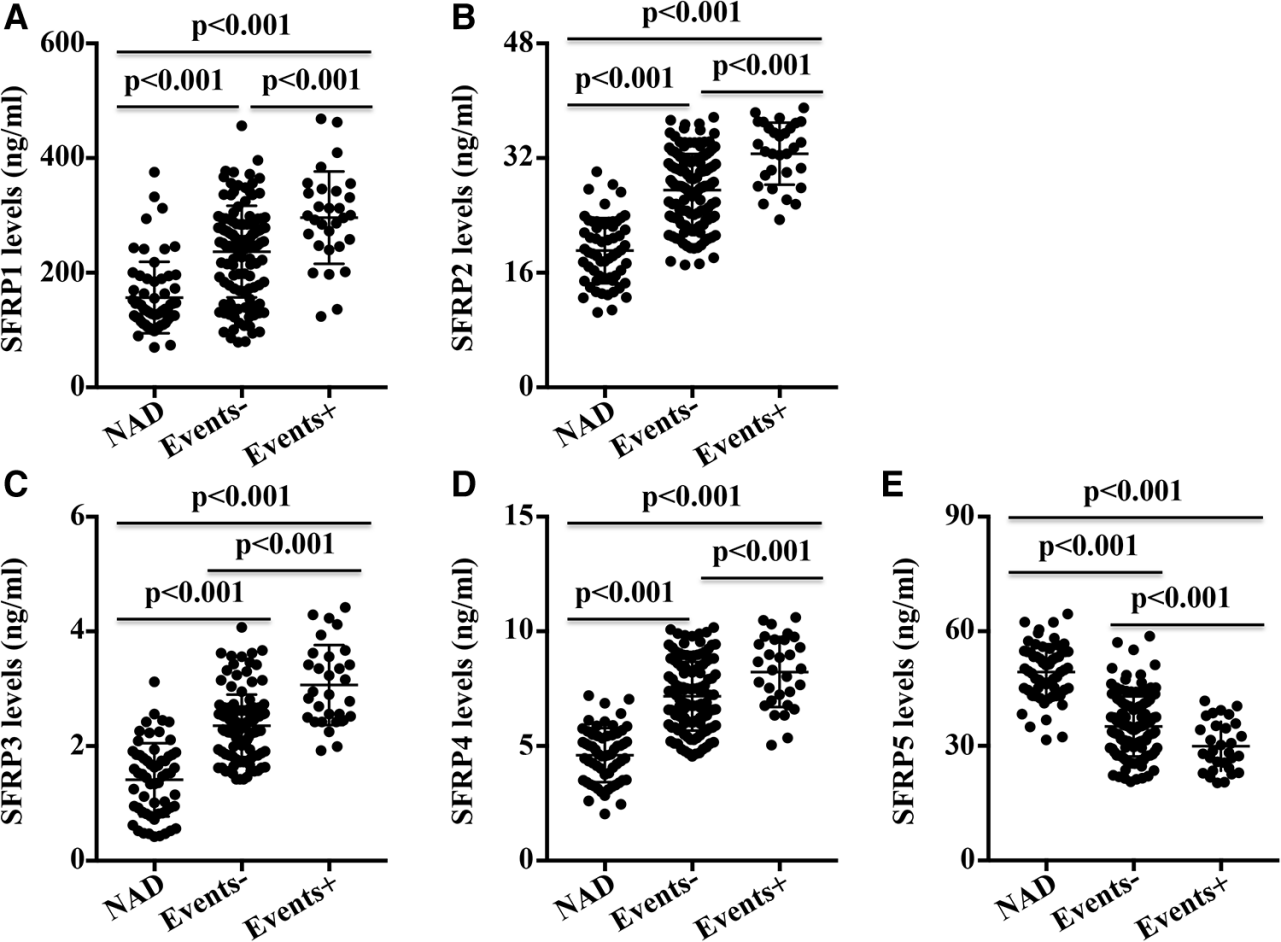
Plasma concentrations of SFRPs in AD patients with (events+) or without (events−) end-point events were detected. (**A**) SFRP1, (**B**) SFRP2, (**C**) SFRP3, (**D**) SFRP4, and (**E**). SFRP5 in the NAD group, events− group, and events+ group was analyzed.

Compared with the AD patients without end-point events, the AD patients with end-point events exhibited higher SFRP1, SFRP2, SFRP3, and SFRP4 levels and lower SFRP5 concentrations (Figures 2A–E). The SFRP1, SFRP2, SFRP3, and SFRP4 levels in the AD patients with or without end-point events were increased compared with the NAD group, while the SFRP5 levels were reduced (Figures 2A–E).

### Effects of SFRPs on prognosis in acute AD patients

**Figure 3:**
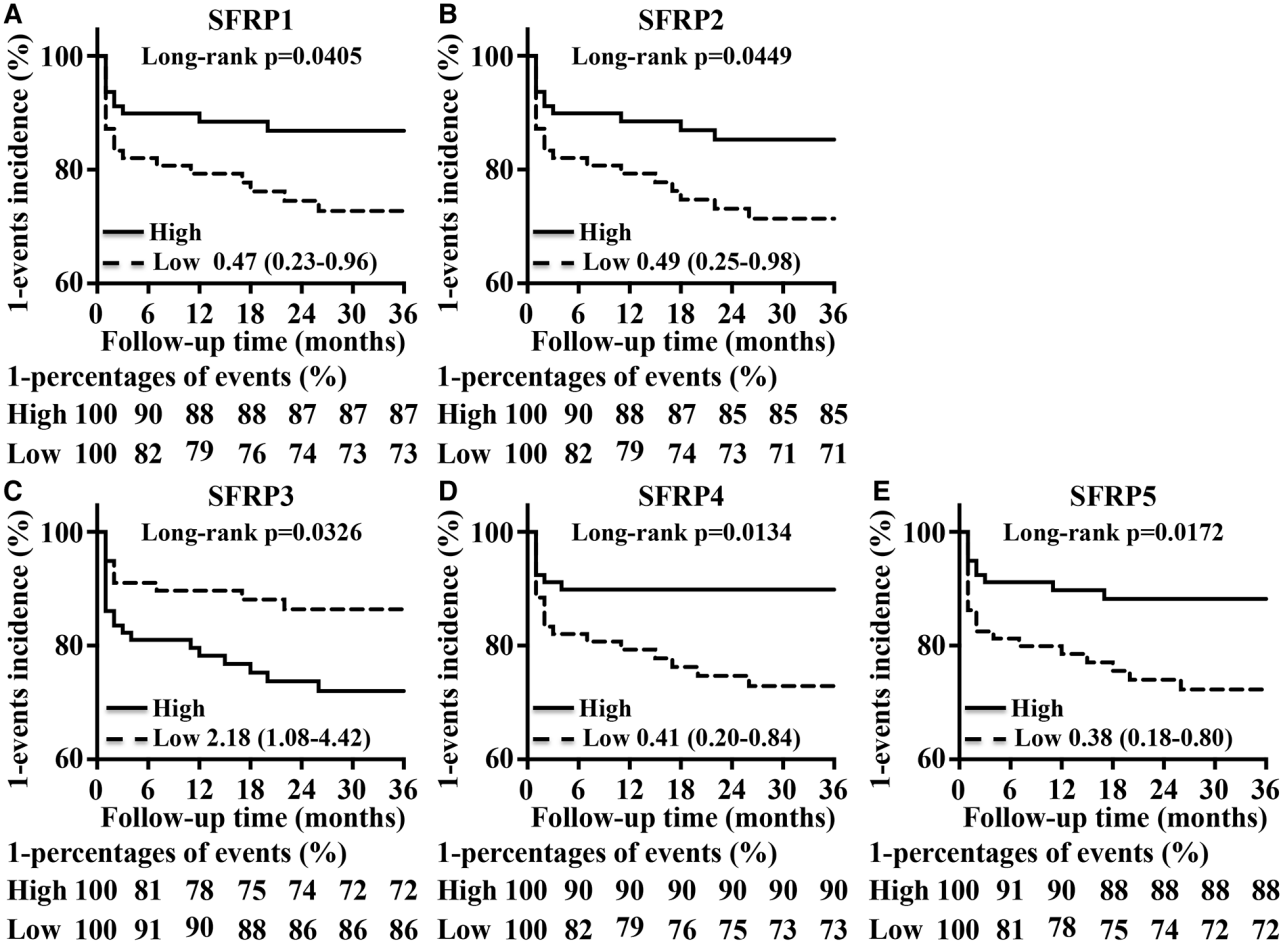
Effects of SFRPs on prognosis in AD patients. The incidence of end-point events in the high group and low group of (**A**) SFRP1, (**B**) SFRP2, (**C**) SFRP3, (**D**) SFRP4, and (**E**). SFRP5 in AD patients was analyzed by the Kaplan–Meier method and log-rank test.

To evaluate prognosis, the AD patients were divided into a high group and a low group based on the median SFRP levels, and the results showed that the group with high levels of SFRP1, SFRP2, SFRP4, or SFRP5 exhibited a better prognosis than the low group (Figures 3A,B,D,E). The high SFRP3 group had a worse prognosis (Figure 3C).

### Effects of SFRPs on the occurrence of endpoint events in acute AD patients

**Figure 4:**
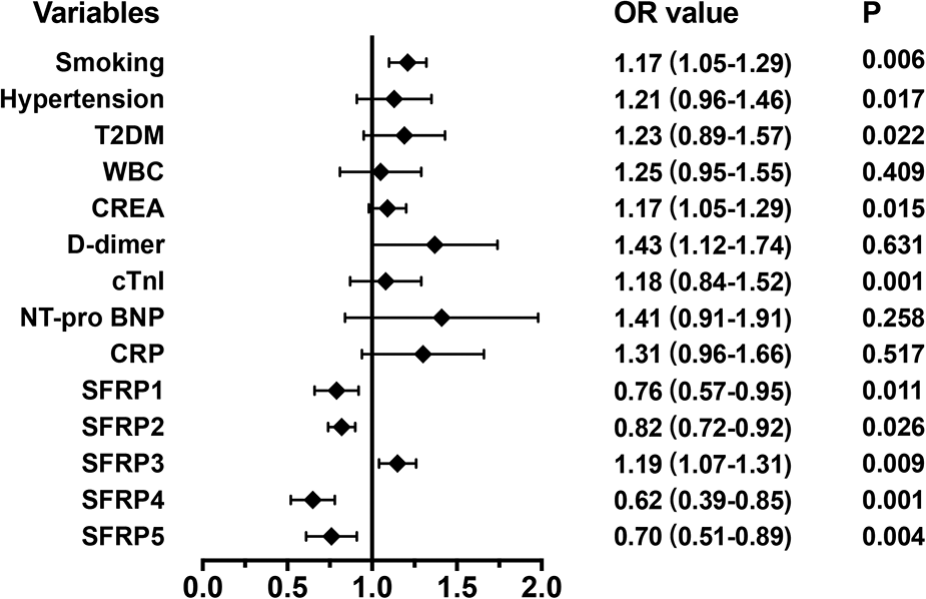
Effects of SFRPs on the occurrence of end-point events were analyzed using binary logistic regression analysis.

To analyze the effect of SFRPs on the occurrence of endpoint events, binary logistic regression analysis was performed. The results showed that smoking and SFRP3 increased the occurrence of endpoint events, while SFRP1, SFRP2, SFRP4, and SFRP5 decreased the occurrence (Figure 4).

## Discussion

In this study, the expression levels of all SFRP members in acute AD patients were examined, and their effects on the prognosis of AD were analyzed. The results showed that the expression of SFRP1, SFRP2 and SFRP4 was increased in AD patients. The results showed that high levels were beneficial to the prognosis, despite decreased concentrations of SFRP5, and high levels of SFRP5 indicated a good prognosis. Although SFRP3 levels were increased, high levels indicated a poor prognosis. This study suggests that as members of the same adipokine family, the expression of SFRPs in AD and their effect on prognosis are not the same.

SFRP1 was the first SFRP family member discovered and is expressed in most tissues and organs. Previous studies found that SFRP1 is involved in downstream signal regulation by regulating the Wnt pathway, and Wnt3 is the main downstream signaling pathway (7). The regulatory effect of SFRP1 on downstream Wnt3 signaling depends on the microenvironment of the body. SFRP1 inhibits Wnt3a activity during the development of the dorsal ganglion neural tube while enhancing Wnt3a activity in L cells (17, 18). Previous studies have found that SFRP1 can significantly reduce cardiac fibrosis and delay the deterioration of cardiac function after acute myocardial infarction (8, 9). In addition, SFRP1 was found to significantly promote tubular formation and angiogenesis *in vitro* (8, 19). These beneficial effects make it possible to treat nonsurgical revascularization patients with angiotherapy, especially in patients with severe coronary artery stenosis who are not candidates for percutaneous coronary intervention (PCI). Our study found that SFRP1 was increased in AD patients, high SFRP1 indicated a better prognosis, and SFRP1 was negatively correlated with the occurrence of the end-point time. These studies suggest that SFRP1 plays a protective role in cardiovascular diseases associated with a variety of different microenvironments, which seems inconsistent with the previous conclusions. One possible reason is that Wnt3 does not mediate all the biological effects of SFRP1, and other pathways, such as P38 and Rac-1, may also play important roles.

SFRP2 is widely expressed and detected in the aorta, although it is not expressed in the heart. SFRP2 exerts its biological effects mainly through Wnt3: not only through wnt3a but also through Wnt3p (20). Similar to SFRP1, the regulatory role of SFRP2 on downstream Wnt3 signals also depends on the microenvironment. SFRP2 plays the same role as SFRP1 in the development of dorsal ganglion neural tubes and L cells (17, 18). In addition, SFRP2 enhances the nuclear translocation of the human embryonic kidney by enhancing the Wnt3p pathway (20). SFRP2 also has a strong protective effect on hypoxia-induced myocardial cell injury in acute myocardial infarction by activating the Wn3a pathway and increasing the expression of MMP2 and MMP9, and its mechanism is highly similar to that of SFRP1 (7, 10, 11). The difference is that SFRP1 mainly regulates angiogenesis by regulating both endothelial cells and vascular smooth muscle cells, while SFRP2 mainly acts on endothelial cells and promotes endothelial tube branching (10). In our current study, we found that the expression trends of SFRP2 were consistent with those of SFRP1 in acute AD patients, and the patients with high levels of SFRP2 showed fewer end-point events and a better prognosis. These results indicate that the role of SFRP2 in patients with AD is consistent with that of SFRP1, and they may have a special, undetermined connection in AD.

SFRP3 is also widely expressed, and the highest expression is in the spleen, suggesting that it may be related to the immune/inflammatory response (21). There are few studies on SFRP3 in cardiovascular diseases. Ueland et al. found that SFRP3 was elevated in patients with acute coronary syndrome, and high SFRP3 levels indicate poor prognosis (13). Askevold et al. reported a very interesting phenomenon in which CHF patients were divided into three groups according to the SFRP3 level, and the middle SFRP3 group showed a better prognosis than the low SFRP3 and high SFRP3 groups, suggesting that CHF patients who maintain SFRP3 at appropriate levels will benefit more than those who have high or low levels of SFRP3 (12). Our study found that acute AD patients had elevated levels of SFRP3, while high levels of SFRP3 suggested a poor prognosis and more end-point points. Unfortunately, the sample size was not large enough; therefore, we could not divide the patients into more groups to determine the optimal SFRP3 level of AD patients.

SFRP4 is moderately expressed in the cardiovascular system and plays an important role in atherosclerosis (AS)-related CAD (7). Our previous study found that SFRP4 played an antiatherogenic role via downstream Wnt1 signaling, while other researchers reported that it was involved in AS-related CAD through the Akt pathway (7, 15). Our previous studies confirmed that SFRP4 expression was increased in CAD and originated mainly from epicardial adipose tissue (EAT), which is a special type of perivascular adipose tissue that can secrete anti-inflammatory factors, inhibit the differentiation of proinflammatory immune cells, and maintain the normal function of blood vessels (8, 22, 23). Studies have found that the transplantation of healthy EAT inhibited the AS process, while the transplantation of diseased EAT played the opposite role (24–26). These seemingly contradictory results are confusing. With further study, researchers found that immune cell infiltration in the EAT of CAD patients was significantly increased and masked the protective effect of healthy EAT (10, 20, 21). In this study, we found that SFRP4 was elevated in AD patients and had prognostic benefit. Combined with the previous studies above, we hypothesized that SFRP4 may be derived from the immune cells within the EAT and participate in the AD process by regulating the inflammatory response.

SFRP5 can also be expressed in the circulatory system. Similar to SFRP4, SFRP5 is secreted by the EAT and visceral fat, and its main downstream signal is Wnt5a (7, 27, 28). Studies have confirmed that SFRP5 is involved in the process of CAD, and both the Wnt5a pathway and JNK pathway are downstream signals. Studies have found that the expression of SFRP5 is decreased in CAD, and its levels are negatively correlated with the severity of CAD and multiple risk factors (28, 29). Our study found that SFRP5 was the only member with decreased expression in AD patients, and high SFRP5 levels suggested a better prognosis.

In conclusion, we examined the effects of SFRPs on the prognosis of AD patients in this study and found that high levels of SFRP1 and SFRP2 may reduce the occurrence of end-point events and have a beneficial effect on prognosis, while high SFRP3 levels may increase adverse events and lead to a poor prognosis. However, a large number of studies have demonstrated that the type of AD, whether undergo surgery, and the time from onset to surgery are the important factors affecting the prognosis of AD. In this study, due to the small sample size, AD patients were not divided into different groups base on these factors and observe the effects of SFRPs on prognosis, respectively. Therefore, whether the effects of SFRPs on prognosis of AD patients were affected by the above factors, although these factors do not differ in the patients with or without end-time points. This is a deficiency of our research that requires further confirmation from more research.

## Data Availability

The raw data supporting the conclusions of this article will be made available by the authors, without undue reservation.
